# Quantitative analysis of the effects of nicotinamide phosphoribosyltransferase induction on the rates of NAD^+^ synthesis and breakdown in mammalian cells using stable isotope-labeling combined with mass spectrometry

**DOI:** 10.1371/journal.pone.0214000

**Published:** 2019-03-15

**Authors:** Nobumasa Hara, Harumi Osago, Mineyoshi Hiyoshi, Mikiko Kobayashi-Miura, Mikako Tsuchiya

**Affiliations:** Department of Biochemistry, Shimane University Faculty of Medicine, Izumo, Shimane, Japan; Macquarie University, AUSTRALIA

## Abstract

NAD^+^ is mainly synthesized from nicotinamide (Nam) by the rate-limiting enzyme Nam phosphoribosyltransferase (Nampt) and degraded to Nam by NAD^+^-degrading enzymes in mammals. Numerous studies report that tissue NAD^+^ levels decrease during aging and age-related diseases and suggest that NAD^+^ replenishment promotes healthy aging. Although increased expression of Nampt might be a promising intervention for healthy aging, forced expression of Nampt gene, inducing more than 10-fold increases in the enzyme protein level, has been reported to elevate NAD^+^ levels only 40–60% in mammalian cells. Mechanisms underlying the limited increases in NAD^+^ levels remain to be determined. Here we show that Nampt is inhibited in cells and that enhanced expression of Nampt activates NAD^+^ breakdown. Combined with the measurement of each cell’s volume, we determined absolute values (μM/h) of the rates of NAD^+^ synthesis (R_S_) and breakdown (R_B_) using a flux assay with a ^2^H (D)-labeled Nam, together with the absolute NAD^+^ concentrations in various mammalian cells including primary cultured cardiomyocytes under the physiological conditions and investigated the relations among total cellular Nampt activity, R_S_, R_B_, and the NAD^+^ concentration. NAD^+^ concentration was maintained within a narrow range (400–700 μM) in the cells. R_S_ was much smaller than the total Nampt activity, indicating that NAD^+^ synthesis from Nam in the cells is suppressed. Forced expression of Nampt leading to 6-fold increase in total Nampt activity induced only a 1.6-fold increase in cellular NAD^+^ concentration. Under the conditions, R_S_ increased by 2-fold, while 2-fold increase in R_B_ was also observed. The small increase in cellular NAD^+^ concentration is likely due to both inhibited increase in the NAD^+^ synthesis and the activation of its breakdown. Our findings suggest that cellular NAD^+^ concentrations do not vary dramatically by the physiological fluctuation of Nampt expression and show the tight link between the NAD^+^ synthesis and its breakdown.

## Introduction

**Fig 1:**
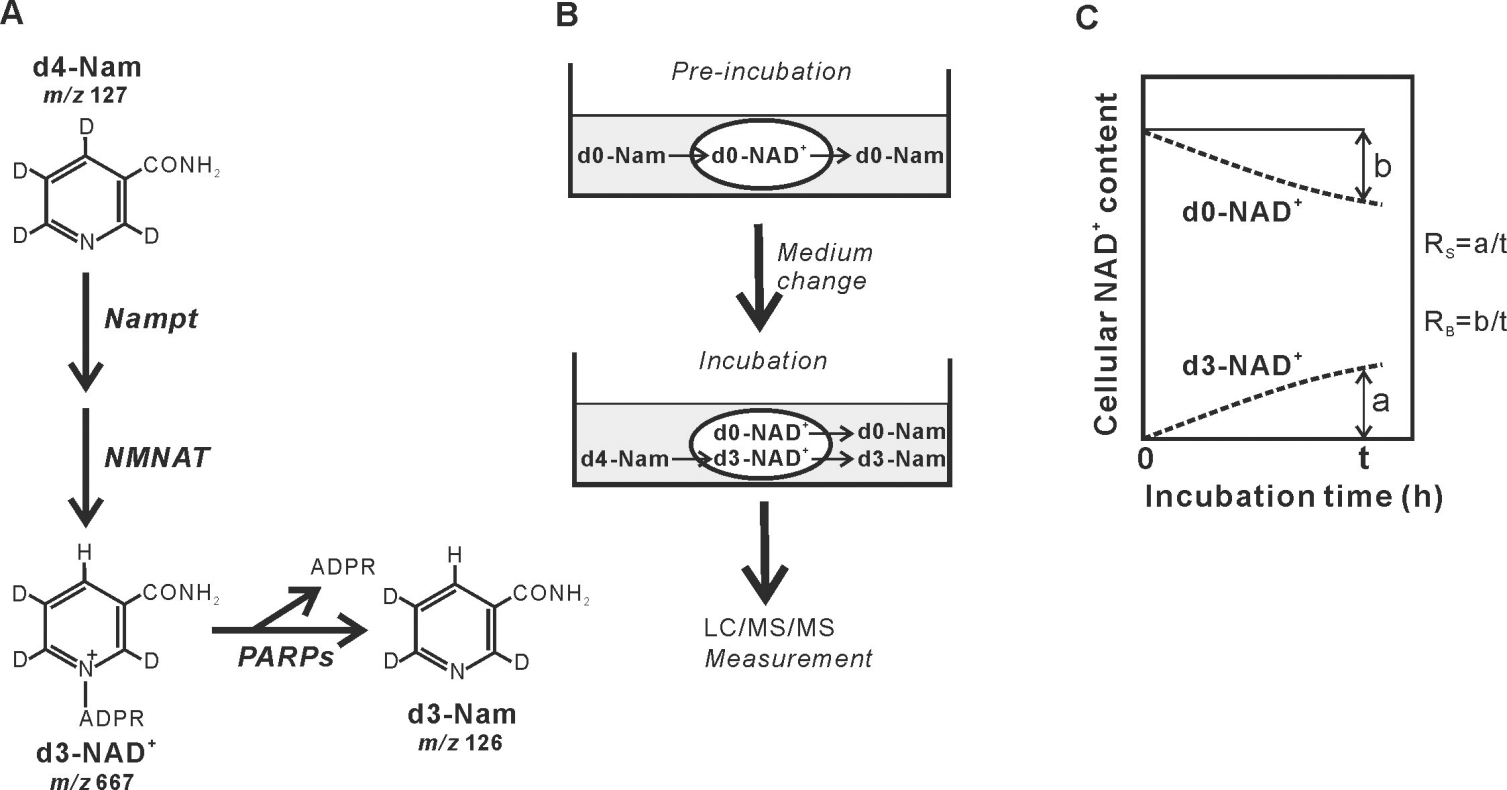
Procedure for determining the rates of NAD^+^ synthesis and breakdown with the deuterium-labeled Nam. (A) Cellular synthesis of labeled NAD^+^ and Nam from d4-Nam. The structures of d4-Nam, formed NAD^+^, and its breakdown product are shown together with their *m/z* values. Key enzymes in the metabolism of NAD^+^ are also shown. NMNAT, Nam mononucleotide adenylyltransferase; ADPR, ADP-ribose. (B) Experimental protocol for determination of the rates of NAD^+^ synthesis and breakdown using LC/MS/MS with d4-Nam. Cells were pre-incubated over night in the medium containing unlabeled Nam (d0-Nam) and then the medium was changed to that containing the same concentration of d4-Nam for the indicated times. After the incubation, NAD^+^ and Nam were extracted from cells and their incubation medium and were quantified by LC/MS/MS. (C) Definition of parameters representing the rates of NAD^+^ synthesis (R_S_) and breakdown (R_B_). R_S_ and R_B_ were calculated from the amounts of d3-NAD^+^ appeared (a) and d0-NAD^+^ decreased (b) during a given incubation time (t).

NAD^+^, a coenzyme in numerous cellular redox reactions in mammals, is mainly synthesized from nicotinamide (Nam) by the rate-limiting enzyme Nam phosphoribosyltransferase (Nampt) through the salvage pathway [1, 2] and degraded to Nam by NAD^+^-degrading enzymes such as poly(ADP-ribose) polymerases (PARPs) [3] (Fig 1A). It is well known that NAD^+^ serves as an essential cofactor for the protein deacetylases sirtuins (SIRTs) [4, 5]. Increasing the activity of SIRTs has been reported to exert protective effects against age-related functional decline and diseases such as metabolic syndrome, neurodegeneration, and cancer [4, 6–8]. Attention is currently focused on physiological and pharmacological interventions boosting cellular NAD^+^ levels to promote healthy aging [9, 10]. The NAD^+^-boosting interventions targeting its synthesis may include increased expression of Nampt. However, enhanced expression of Nampt gene leading to 10-20-fold increases in the enzyme protein has been reported to elevate the NAD^+^ levels only 40–60% in mammalian culture cells and tissues [11–14]. This relatively modest increase in NAD^+^ levels has been proposed to be due to a strong feedback inhibition of Nampt by cellular NAD^+^ [12, 14] and/or activation of NAD^+^ breakdown [14], but not yet demonstrated.

In the present study, we investigated whether Nampt is indeed inhibited in cells and whether enhanced expression of Nampt activates NAD^+^ breakdown. For this purpose, it is essential to obtain all of total Nampt activity in cells, the rates of the NAD^+^ synthesis (R_S_) and breakdown (R_B_), and NAD^+^ concentration in cells. To measure R_S_ and R_B_, two recent studies have determined the incorporation of ^2^H (D)-labeled precursor [2, 4, 5, 6-D_4_]Nam (d4-Nam) into cellular NAD^+^ [15, 16]. We here set optimal concentrations of the labeled Nam and labeling times for accurate measurement of R_S_ and R_B_ in common mammalian cell lines under the physiological conditions and then examined effects of the induced expression of Nampt on R_S_ and R_B_, as well as cellular NAD^+^ concentration. To obtain their absolute values, the volume of each cell was determined in parallel. Measurement of R_S_ and R_B_ together with total Nampt activity in the Nampt-over-expressed cells revealed that R_S_ is much smaller than total Nampt activity and that increases in R_S_ are much smaller than those in the Nampt activity, suggesting the inhibition of Nampt and/or insufficient availability of Nam in the cells. Upon Nampt over-expression, increase in R_B_, dependent on the increase in R_S_, was induced. Consistent with the inhibited increase in R_S_ and the associated increase in R_B_, NAD^+^ concentration was not largely elevated in the cells. As with the induced cells, in other cells examined here including primary cultured cardiomyocytes, we also found that R_S_ is much smaller than total Nampt activity and is balanced with R_B_. NAD^+^ concentration in all these cells was maintained within a narrow range and was much higher than those reported to inhibit Nampt irrespective of cell origins. Our study indicates that cellular NAD^+^ concentration tends to be held constant against fluctuation of Nampt protein levels and provides direct evidence for the tight regulation of cellular NAD^+^ concentrations.

## Materials and methods

### Materials

[2, 4, 5, 6-D_4_]Nam (d4-Nam) was purchased from CDN isotope (Quebec, Canada) and was further HPLC-purified to remove contaminating a significant level of d4-nicotinic acid (NA) (5%) [17]. Nam was purchased from FUJIFILM Wako Pure Chemical Corporation (Osaka, Japan). [*carbonyl*-^14^C]Nam (50 mCi/mmol) was purchased from American Radiolabeled Chemical Inc. (St. Louis, MO, USA). NAD^+^ and 5-phosphoribosyl 1-pyrophosphate (PRPP) were purchased from Oriental Yeast (Tokyo, Japan) and Sigma-Aldrich (St. Louis, MO, USA), respectively. Calibration standards d3-NAD^+^ and d3-Nam were prepared with recombinant NAD^+^ biosynthetic enzymes, as described previously [17]. The concentrations of the labeled standards were determined based on the integrated peak areas of the absorbance at 254 nm compared to those of unlabeled ones on HPLC.

### Cell culture

Human cervical carcinoma HeLa (RCB0007), hepatoma HepG2 (RCB1648), colon carcinoma Caco-2 (RCB0988), embryonic kidney 293T (RCB2202), and mouse myoblast C2C12 (RCB0987) cells were obtained from Riken Cell Bank (Tsukuba, Japan). Human embryonic kidney 293 (JCRB9068), rat hepatoma Fao (89042701), and rat cardiomyocyte H9c2 (CRL-1446) cells were obtained from Japanese Collection of Research Bioresources (JCRB) Cell Bank (National Institutes of Biomedical Innovation, Health and Nutrition, Osaka, Japan), ECACC (Salisbury, UK), and ATCC (Manassas, VA, USA), respectively. These cells were maintained in Eagle’s minimum essential medium (MEM, FUJIFILM Wako Pure Chemical Corporation) containing antibiotics and 10% fetal bovine serum, except for C2C12 and H9c2 cells, which were maintained in Dulbecco’s modified Eagle medium (FUJIFILM Wako Pure Chemical Corporation). Primary cultured rat cardiomyocytes (CMC02) were obtained from COSMO BIO (Tokyo, Japan).

### Inducible expression of Nampt

To achieve inducible expression of human Nampt by Tet-On system, the coding region of the enzyme was subcloned into a tetracycline-responsive element (TRE)-containing vector pTRE-Tight (Clontech, Mountain View, CA, USA). This vector was transiently transfected using PolyFect transfection reagent (Qiagen, Hilden, Germany) into HeLa cells together with pTet-On-Advanced vector (Clontech), which expresses the tetracycline-regulated transactivator and activates expression from TRE promoters in the presence of doxycycline (Dox). Expression was induced by the addition of Dox to the culture medium at a final concentration of up to 1 μg/mL.

### Incubation of cells with the deuterium-labeled Nam

HepG2, Fao, C2C12, H9c2, and the primary cultured cardiomyocytes were seeded on collagen I-coated 12-well plates (BD Biosciences, Bedford, MA, USA). Caco-2, 293, and 293T cells, and HeLa cells were seeded on poly-D-lysine-coated (BD Biosciences) and CELLSTAR (Greiner Bio-One, Frickenhausen, Germany) 12-well plates, respectively. These cells were cultured in 0.95 ml of the custom-made MEM without Nam (Nam-free MEM, Biological Industries, Beit Haemek, Israel), to which indicated concentrations of either d0-Nam or d4-Nam were added together with the dialyzed serum at a final concentration of 0.5%. The cells were pre-cultured with the same concentrations of unlabeled Nam (d0-Nam) and the serum.

### Extraction of NAD^+^ and Nam

For extracting NAD^+^ from the cells, 0.4 ml of 0.5 N perchloric acid was directly added to the well after the removal of culture medium. The media were mixed with one-tenth volume of 5 N perchloric acid to precipitate proteins. After centrifugation of the extracts, the supernatants were collected, neutralized with an equal volume of 1 M ammonium formate, and subjected to LC/MS/MS, as described below. For each experiment, a replicate set of cells was treated identically, but with the same concentration of d0-Nam, and used for cell counting, determination of cell volume, and preparation of lysate from the cells, as described below.

### Cell counting and determination of cell volume

The cells were trypsinized, suspended, and counted using an automated cell counter Scepter (Millipore, Billerica, MA, USA), which also provides a readout of the approximate volume of the counted cells based on the Coulter Principle, as described previously [18], thus allowing to calculate cellular concentrations of NAD^+^.

### Preparation of lysates from culture cells

The cells were collected and sonicated in buffer containing 20 mM Tris-Cl^-^ (pH 7.5), 10% glycerol, and protease inhibitor mixture (Roche Diagnostics, Indianapolis, IN, USA). After centrifugation, the supernatants were subjected to Nampt assay or Western blot analysis, as described below.

### Nampt assay

Cell lysates were incubated with 50 μM [^14^C]Nam and 0.3 mM PRPP in the reaction mixture containing 50 mM Tris-Cl^-^ (pH 7.5), 10 mM MgCl_2_, 2.5 mM DTT, 0.1 mg/ml BSA,1 mM ATP, 147 mM KCl, and 5 mM Pi. After the incubation at 37°C for 1 h, the reaction was terminated by heating in a boiling water for 60 s. Proteins were removed by centrifugation, and the reaction products were separated by a thin layer chromatography on silica gel sheets (Merck, Darmstadt, Germany) as described previously [19], and visualized and quantified using a bio-imaging analyzer FLA-7000 (FUJIFILM, Tokyo, Japan).

### Western blot analysis

Cell lysates were separated on SDS-PAGE, transferred onto Immobilon-P membrane (Millipore), and blocked with 10% EzBlock Chemi (ATTO, Tokyo, Japan). The membranes were incubated with rabbit anti-human Nampt polyclonal antibodies (1:3000 dilution, Sigma-Aldrich, V9139) in Can Get Signal Immunoreaction Enhancer Solution 1 (Toyobo, Osaka, Japan) for 18 h at 4°C followed by horseradish peroxidase-conjugated goat anti-rabbit IgG polyclonal antibodies (1:10000 dilution, MBL, Nagoya, Japan, #458) in Can Get Signal Immunoreaction Enhancer Solution 2 (Toyobo) at room temperature for 3 h. The bound antibodies were detected with Chemi-Lumi One Super (Nacalai Tesque, Kyoto, Japan). Comparable loading of proteins was confirmed using rabbit polyclonal anti β-actin antibodies (1:4000 dilution, MBL, PM053) in Can Get Signal Immunoreaction Enhancer Solution 1.

### LC/MS/MS

LC/MS/MS was performed using Shimadzu HPLC system (Shimadzu, Kyoto, Japan) that was connected to a triple quadrupole tandem mass spectrometer (Sciex API 3000, Applied Biosystems, Foster City, CA, USA) equipped with a turbo ion-spray ionization source operating in the positive mode. The labeled and unlabeled NAD^+^ metabolites were separated on an Atlantis dC18 resin (150 x 2.1 mm i.d. column; 3-μm particle size; Waters, Milford, MA, USA). The mobile phase consisted of 5 mM ammonium formate (solvent A) and methanol (solvent B). The percentage of solvent B was changed lineally as follows: 0 min, 0%; 10 min, 70%; 15 min, 70%; 15.1 min, 0%; and 20 min, 0%. The flow rate was 0.15 ml/min. Precursor ion spectra of NAD^+^ and Nam were generated by scanning the ranges of *m/z* from 650 to 700 and from 100 to 150, respectively. Each of the metabolites was subjected to collision-induced dissociation to obtain product ions. Product ion spectra were generated by scanning the ranges of *m/z* from 80 to 700 for NAD^+^ and from 70 to 130 for Nam. Quantification of the labeled metabolites was performed by selected reaction monitoring (SRM) mode with the mass spectrometer parameters provided in S1 Table. Using labeled calibration standards synthesized *in vitro*, as described above, as well as unlabeled standards, the calibration curves for these compounds were constructed with the SRM transitions indicated. ABI Analyst software (version 1.4) was used for data acquisition and processing. All calibration curves exhibited excellent linearity (S1 Fig). The lower limits of quantification were 0.1–0.4 pmol for these compounds (S1 Table).

Measurements were also carried out using a LCMS-8030 triple quadrupole mass spectrometer (Shimadzu) equipped with a Shimadzu Prominence FPLC system and the Atlantis dC18 resin. The mobile phase solvents and the flow rate were as described above. Percent of solvent B was changed lineally as follows: 0 min, 2%; 3 min, 20%; 6 min, 70%; 11 min, 70%; 11.1 min, 2%; and 17 min, 2%. The SRM parameters for LC/MS/MS are provided in S2 Table. Shimadzu LabSolutions software (version 5.60SP2) was used for data acquisition and processing.

### Statistical analysis

Statistical data are expressed as the mean ± S.D. of *n* experiments.

## Results

### Definition of the rates of NAD^+^ synthesis and breakdown

In this study, we determined R_S_ and R_B_ in various mammalian cell lines established from cancerous and non-cancerous origins together with primary cultured rat cardiomyocytes. To measure R_S_ and R_B_, cells pre-incubated with unlabeled Nam (d0-Nam) are then incubated with d4-Nam (Fig 1B). Based on the continual synthesis and breakdown of NAD^+^ [15, 16], d3-NAD^+^ is produced in the cell [17] while unlabeled NAD^+^ (d0-NAD^+^) is degraded, depending on the incubation time (Fig 1C). We here defined R_S_ and R_B_ as *a*/*t* and *b*/*t*, respectively, where *t*, *a*, and *b* are respectively the incubation time, the amount of d3-NAD^+^ newly appeared, and that of d0-NAD^+^ decreased during the incubation, as schematically shown in Fig 1C. Total cellular NAD^+^ contents are the sum of the amounts of d3-NAD^+^ and d0-NAD^+^.

### Conditions for accurate determination of the rates of NAD^+^ synthesis and breakdown

Because of the deuterium isotope effects on enzyme-catalyzed reaction rates [20, 21], the rate of NAD^+^ synthesis from d4-Nam may not be identical to that from the unlabeled precursor, and the resultant labeled NAD^+^ may also be metabolized with different rates by cellular NAD^+^-degrading enzymes compared with unlabeled NAD^+^. Furthermore, Nam produced from NAD^+^ by the NAD^+^-degrading enzymes during the incubation can be reincorporated into NAD^+^, underestimating the rates [22]. Thus, methodological requirements for accurate measurement of the rates with d4-Nam are as follows: (1) d4-Nam should exhibit similar bioavailability to d0-Nam, and d3-NAD^+^ synthesized also behaves similarly to d0-NAD^+^, and (2) the recycling of Nam derived from the NAD^+^ degradation for NAD^+^ synthesis is so small that it can be ignored.

We first compared the bioavailability of d4-Nam as well as the d3-NAD^+^-degrading product d3-Nam with that of d0-Nam and found that total cellular NAD^+^ contents after the incubation with d0-Nam, d4-Nam, or d3-Nam were almost similar, irrespective of the precursors (S2A Fig). In addition, upon treatment with a PARP activator *N*-methyl-*N’*-nitro-*N*-nitrosoguanidine of cells pre-incubated with d4-Nam or d0-Nam, roughly the same amount of Nam was produced from the cells (S2B Fig). These observations demonstrate that d4-Nam and d3-Nam exhibit similar bioavailability to d0-Nam and that d3-NAD^+^ and d0-NAD^+^ are similarly degraded by PARPs, and thus, the d4-Nam can be used to determine R_S_ and R_B_ at least cellular levels. We further observed almost the same ATP contents after incubating cells with d4-Nam or d0-Nam (S2C Fig), excluding significant effects of the labeling with deuterium on cellular redox reactions involving NAD^+^ and NADH, and again supporting the use of d4-Nam to determine R_S_ and R_B_.

**Fig 2:**
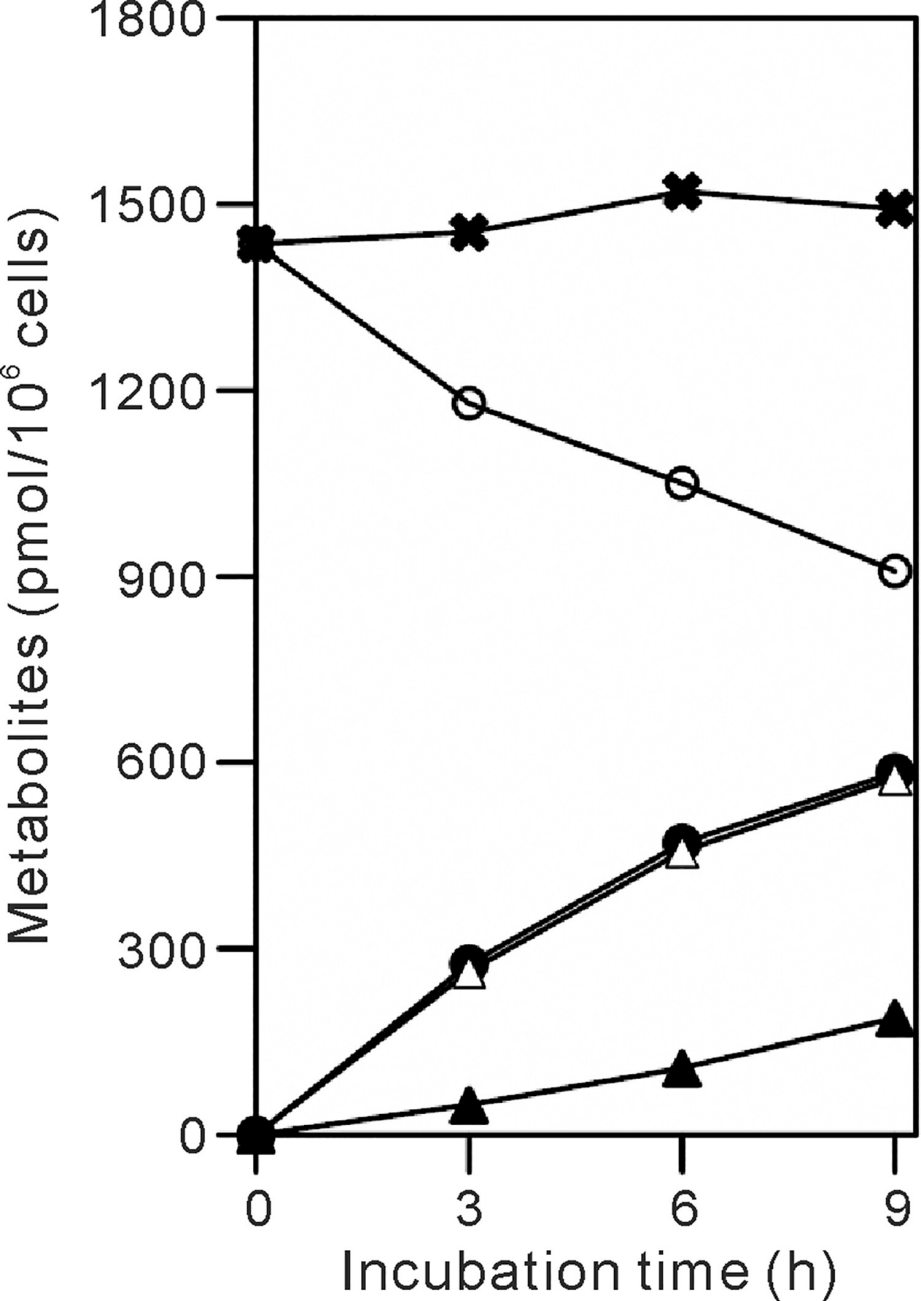
Determination of the rates of NAD^+^ synthesis and breakdown with d4-Nam. HepG2 cells (4.04 x 10^5^ cells) were cultured in d0-Nam-free MEM supplemented with 2 μM d4-Nam for the indicated times. After the incubation, isotopomers of NAD^+^ in the cells and Nam in the culture medium were extracted, separated by HPLC, and quantified. Amounts of d3- (*filled circles*) and d0-NAD^+^ (*open circles*), and d3- (*filled triangles*) and d0-Nam (*open triangles*) are plotted, as a function of incubation times. The total amounts of NAD^+^ (*crosses*) consisting of d3- and d0-NAD^+^ are also shown.

**Fig 3:**
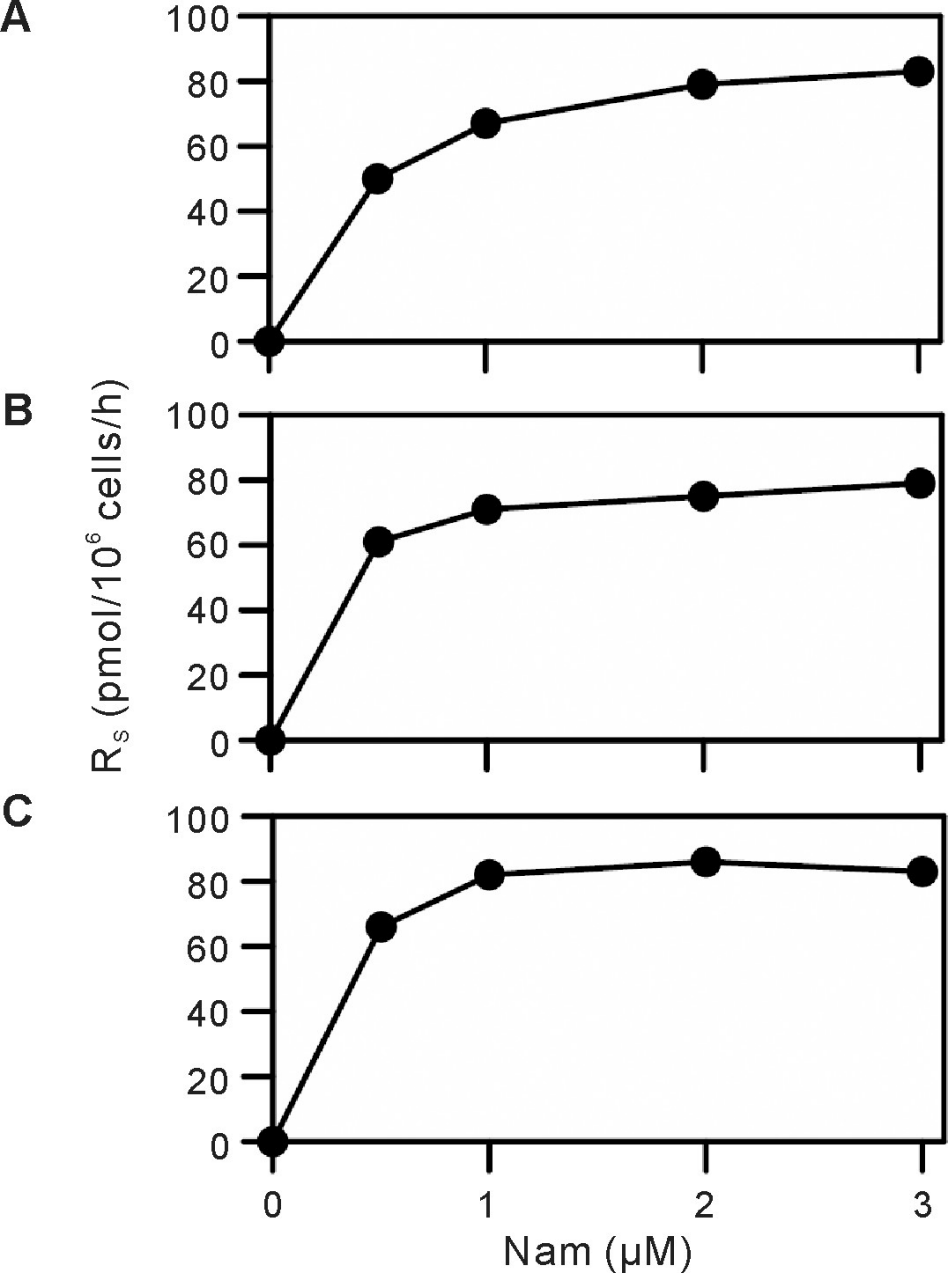
The effect of concentrations of d4-Nam in medium on R_S_. Fao (A), HepG2 (B), and HeLa (C) cells were incubated with d0-Nam-free MEM supplemented with indicated concentrations of d4-Nam for 3 h. After the incubation, d3-NAD^+^ was quantified to determine R_S_ (*circles*).

We next examined the recycling of Nam derived from the NAD^+^ degradation for NAD^+^ synthesis. Cells were incubated with d4-Nam for various times and quantified the labeled metabolites together with unlabeled ones in the cells and medium. When HepG2 cells were incubated with 2 μM d4-Nam for 0, 3, 6, and 9 h, the amount of d3-NAD^+^ increased whereas that of d0-NAD^+^ decreased, depending on the incubation time (Fig 2). Along with the degradation of NAD^+^, d3-Nam as well as d0-Nam was detected in the culture medium (Fig 2), together with d4-Nam (see below). After the incubation for 3 h, 276 pmol of d3-NAD^+^/10^6^ cells was produced while d0-NAD^+^ was decreased by 258 pmol/10^6^ cells (Fig 2). According to our definition, R_S_ and R_B_ were calculated to 92 and 86 pmol/10^6^ cells/h, respectively. As the sum of the amounts of d3-NAD^+^ and d0-NAD^+^, total NAD^+^ content was 1455 pmol/10^6^ cells at this time (Fig 2). In the medium, 48 pmol of d3-Nam/10^6^ cells and 264 pmol of d0-Nam/10^6^ cells were detected (Fig 2). The percent of the concentrations of d3-Nam, d0-Nam, and the precursor d4-Nam in the culture medium at this time were 1.0, 5.4, and 93.6%, respectively, indicating no significant recycling of d0-Nam and d3-Nam into NAD^+^ when the cells were incubated for 3 h, and thus, accurate determination of the rates under the conditions. Because we found that maximum rate of the synthesis was observed at micromolar concentrations of Nam (Fig 3 and S3 Fig), R_S_ and R_B_ were determined by incubating the cells with 2 μM d4-Nam for 3 h in the following experiments.

### R_S_ is much smaller than total Nampt activity in mammalian cells

**Fig 4:**
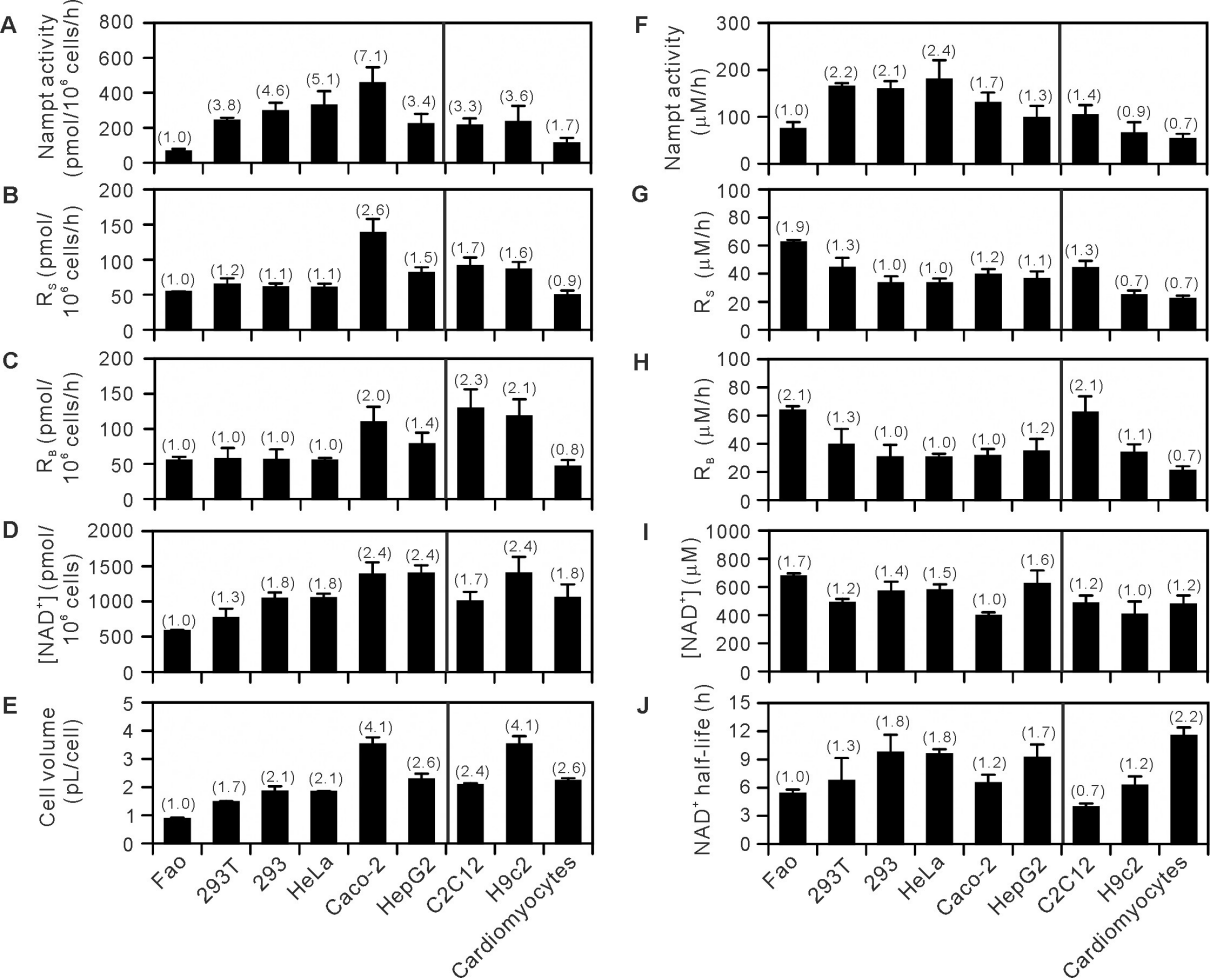
Absolute values of NAD^+^ level, R_S_, R_B_, total Nampt activity in mammalian cells. Fao, 293T, 293, HeLa, Caco-2, HepG2, C2C12, H9c2, and the primary cultured cardiomyocytes were incubated in d0-Nam-free MEM supplemented with 2 μM d4-Nam for 3 h. After the incubation, d3- and d0-NAD^+^ were quantified to determine R_S_ (B) and R_B_ (C) as well as total NAD^+^ content (D). Total Nampt activity was determined in cells incubated with 2 μM d0-Nam for 3 h (A). The suspended cells were counted using an automated cell counter, which also provides a readout of the approximate volume of the counted cells (E). Total Nampt activity, R_S_, R_B_, and total NAD^+^ content were normalized to cell volume to obtain absolute values (F-I). Half-life of cellular NAD^+^ (J) was calculated from R_B_ and cellular NAD^+^ concentration. Data shown are results from 3, 3, 4, 3, 5, 3, 4, 5, and 3 independent experiments for Fao, 293T, 293, HeLa, Caco-2, HepG2, C2C12, H9c2, and the primary cultured cells, respectively. The smallest values within those obtained from Fao, 293T, 293, HeLa, Caco-2, and HepG2 cells in each panel were set to 1.0 and relative values are shown in parentheses.

To investigate whether Nampt is inhibited in cells, we determined R_S_ in 6 different common mammalian cancerous cell lines Fao, 293T, 293, HeLa, Caco-2, and HepG2 cells under the conditions established above, and compared the obtained R_S_ with total Nampt activity in the cells. As shown in Fig 4A, markedly varying total Nampt enzyme activities were observed in these cells. For example, the Nampt activity in Caco-2 cells was 7-fold higher than that in Fao cells. Differences in cell volume (Fig 4E) can in part account for the substantial differences that exist among the cells (Fig 4A). Indeed, after normalized to cell volumes, the Nampt activities were found to be within 2.4-fold (Fig 4F). We thus normalized all of total Nampt activity, R_S_, R_B_, and NAD^+^ levels in the cells to their cell volumes (Fig 4F–4I). In Fig 4F and 4G, R_S_ was compared with the Nampt activity. In HeLa cells, R_S_ was 33 μM/h and much lower than the Nampt activity (180 μM/h). Even in cells with the highest R_S_ among the cells examined (62 μM/h, Fao), the rate was lower than total Nampt activity (74 μM/h).

**Fig 5:**
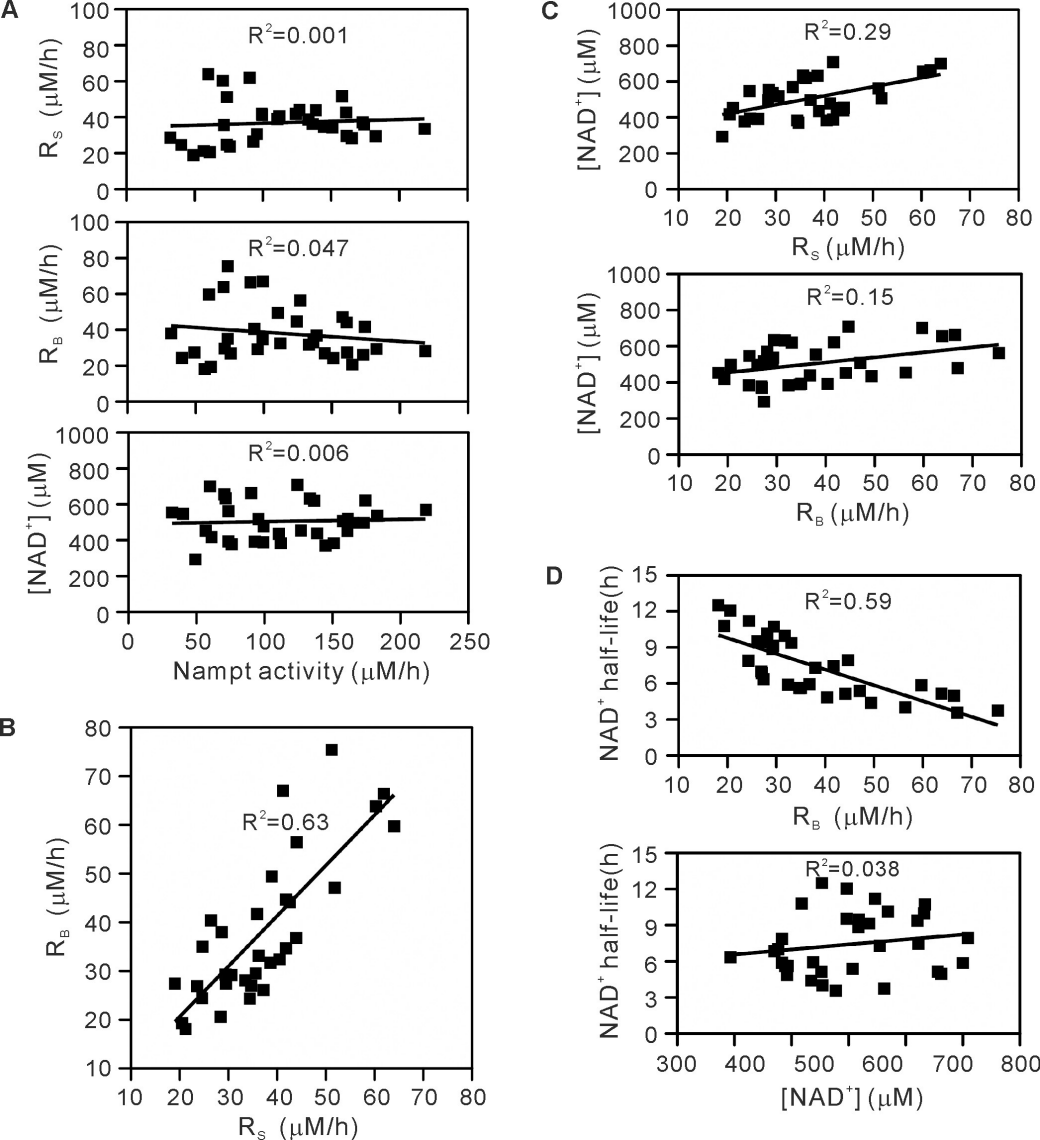
Relationship among total Nampt activity, R_S_, R_B_, cellular NAD^+^ concentration, and calculated NAD^+^ half-life. Data in Fig 4 were re-plotted. (A) Relation of R_S_, R_B_, and the NAD^+^ concentration to total Nampt activity. (B) Relation between R_S_ and R_B_. (C) Relation of the NAD^+^ concentration to R_S_ or R_B_. (D) Relation of NAD^+^ half-life to R_B_ or the NAD^+^ concentration. Determination coefficience (R^2^) is also indicated.

To test whether such a relationship between the rate of NAD^+^ synthesis and the Nampt activity obtained in the common cell lines established from cancerous origins can be extended to those established from non-cancerous origins and primary cultured cells, we examined two additional cell lines, mouse myoblast C2C12 and rat cardiomyocyte H9c2 cells, and primary cultured rat cardiomyocytes. As shown in Fig 4F and 4G, R_S_ was again lower than the total Nampt activity in these cells, indicating that the rate of NAD^+^ synthesis is much smaller than total Nampt activity in mammalian cells. Re-plot of R_S_ against the total Nampt activity in all these cells revealed that the rate of NAD^+^ synthesis is not directly proportional to the Nampt activity (Fig 5A).

**Fig 6:**
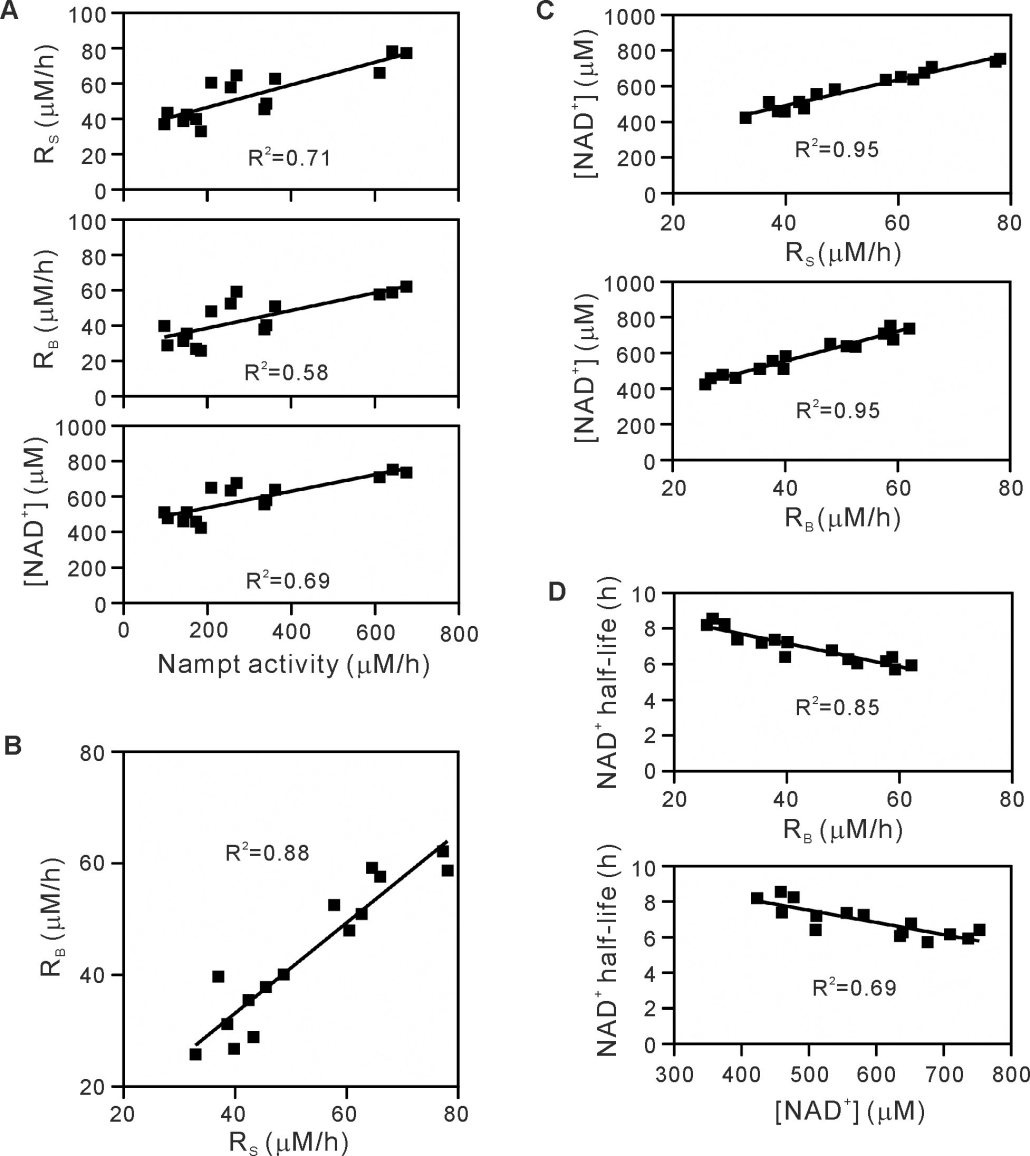
The effects of induced expression of Nampt on R_S_, R_B_, cellular NAD^+^ concentration, and half-life of NAD^+^. Nampt expression was induced in HeLa cells by incubating with 0.1, 0.2, 0.3, or 1.0 μg/mL of Dox. (A) Relation of R_S_, R_B_, and cellular NAD^+^ concentration to total Nampt activity. (B) Relation between R_S_ and R_B_. (C) Relation of cellular NAD^+^ concentration to R_S_ or R_B_. (D) Relation of NAD^+^ half-life to R_B_ or the NAD^+^ concentration. Determination coefficience (R^2^) is also indicated.

To further investigate a relation between R_S_ and total Nampt activity, Nampt was over-expressed using the Dox-inducible gene expression system in HeLa cells (S4 Fig). As shown in Fig 6A, induction of Nampt increased total Nampt activity from 100 to 600 μM/h. Under the conditions, R_S_ increased from 40 to 80 μM/h, and was again much smaller than the total Nampt activity in the cells, irrespective of the Nampt induction levels (Fig 6A). The 6-fold increase in total Nampt activity increased R_S_, but only by 2-fold (Fig 6A). This was not due to the depletion of PRPP in the Nampt-over-expressed cells. HeLa cells express endogenous nicotinic acid (NA) phosphoribosyltransferase, a first enzyme in the synthesis of NAD^+^ from NA [19], which forms NA mononucleotide from NA and PRPP. The addition of 5 μM NA to these Nampt-induced cells further elevated cellular NAD^+^ concentration to 1065 ± 45 μM (n = 5), excluding the possibility that the cellular pool of PRPP had been severely depleted in the Nampt-over-expressed cells. Treatment with the same concentration of Dox of HeLa cells without transfection did not affect R_S_, R_B_, or cellular NAD^+^ concentration (S5 Fig). Furthermore, when HeLa cells transfected with empty vector without Nampt insert were treated with or without Dox, obtained R_S_, R_B_, and cellular NAD^+^ concentration were similar to those in non-transfected cells without Dox (S5 Fig). All these data demonstrate that Nampt is significantly inhibited in the cells.

### R_B_ depends on R_S_

To investigate whether the breakdown of NAD^+^ is tightly linked to its synthesis, R_S_ and R_B_ were compared in various mammalian cells. As shown in Fig 4G and 4H, the rate of NAD^+^ synthesis was similar to that of its breakdown in these cells. A re-plot of the data in Fig 4G and 4H gave a linear relationship between R_S_ and R_B_ in all cells examined (Fig 5B), indicating a balanced synthesis and breakdown of cellular NAD^+^, namely R_S_ = R_B_, and suggesting a tight link between the NAD^+^ synthesis and its breakdown.

Furthermore, the effect of the enhanced synthesis of NAD^+^ on the NAD^+^ breakdown was examined in the Nampt-induced cells. As shown in Fig 6A, under the conditions where 2-fold increase in R_S_ was observed in response to the Nampt induction, R_B_ increased 2-fold from 30 to 60 μM/h. A linear relationship between R_S_ and R_B_ was again observed in the induced cells (Fig 6B). These observations indicate the induction of NAD^+^ breakdown associated with the increased synthesis of this nucleotide. This induction of NAD^+^ breakdown was not due to increased expression of NAD^+^-consuming enzymes PARP1, PARP2, SIRT1, or CD38 [23] (S6 Fig).

### Enhanced expression of Nampt increases cellular NAD^+^ concentration no more than 2-fold

We determined the rates of NAD^+^ synthesis and breakdown together with cellular NAD^+^ concentration in various mammalian cells including the primary cultured cells and Nampt-induced cells. In the Nampt-uninduced cells, cellular NAD^+^ concentration was maintained within 400–700 μM (Fig 4I) and was not directly proportional to the Nampt activity (Fig 5A). However, Nampt induction leading to more than 6-fold increase in Nampt activity elevated NAD^+^ concentration in the cells, but only by 1.6-fold from 500 to 800 μM (Fig 6A). These results indicate that cellular NAD^+^ concentration is maintained within a narrow range and does not increase markedly upon increased expression of Nampt. Both much smaller increase in R_S_ compared with the increase in total Nampt activity (Fig 6A) and the accompanied increase in R_B_ (Fig 6A and 6B) explain the absence of marked increase in NAD^+^ concentration in the Nampt-induced cells. As shown in Fig 6C, cellular NAD^+^ concentration increased with R_S_ and R_B_ in the Nampt-induced cells, while a increasing trend was observed in the uninduced cells (Fig 5C).

The half-life of cellular NAD^+^ can be estimated from NAD^+^ concentration and R_B_ in the cells, and was calculated to 3.9–11.5 h (Figs 4J and 6D). The half-life, decreased with R_B_ (Figs 5D and 6D, *tops*), was relatively consistent irrespective of cellular NAD^+^ concentration (Figs 5D and 6D, *bottoms*).

## Discussion

In the present study, using stable isotope-labeling experiments combined with mass spectrometry together with cell volume measurement, we demonstrated that cellular NAD^+^ concentration is maintained within a narrow range irrespective of total activity of Nampt in primary cultured rat cardiomyocytes as well as various mammalian cell lines established from cancerous and non-cancerous origins and that forced increase in Nampt expression by 6-fold elevates cellular NAD^+^ concentration no more than 2-fold. Comparison of R_S_ and R_B_ with the total Nampt activity in the Nampt-over-expressed cells revealed possible mechanisms underlying the tight regulation of cellular NAD^+^ concentration against fluctuation of Nampt protein levels. Elucidation of the molecular basis underlying the tight regulation may provide insight into homeostasis of cellular NAD^+^ concentration.

We determined R_S_ in various mammalian cells under the resting conditions and compared R_S_ with total Nampt activity in the cells. We found that in all cells examined R_S_ is much smaller than the total Nampt activity, indicating that NAD^+^ synthesis from Nam in cells is suppressed. Previous *in vitro* study [19] demonstrates that Nampt is subjected to the negative feedback inhibition by cellular levels of NAD^+^, and that the inhibition is due to decrease in affinity for substrates of Nampt Nam and PRPP. In the presence of 500 μM NAD^+^, the cellular concentration we obtained here, the *K*_m_ value for Nam in the Nampt reaction should increase from 1 to 25 μM [19]. If this is the case, increasing Nam concentration in the medium would significantly increase R_S_. However, we observed maximum R_S_ at 1 μM Nam in the medium and no significant increase in R_S_ by further increase in the concentration of Nam. Taken together, Nam availability in the cells might not be sufficient for Nampt to exhibit full activity. Under such an insufficient availability of Nam, forced increase in Nampt protein levels in cells would not increase R_S_ further. Indeed, in the Nampt-over-expressed cells, R_S_ increased only 2-fold in response to the 6-fold increase in the total Nampt activity, indicating that increase in R_S_ is suppressed and that Nampt activity determined with cell lysates does not simply define R_S_. In our experiments, Nampt was over-expressed in HeLa cells with endogenous NA phosphoribosyltransferase, which forms an intermediate in NAD^+^ synthesis from the substrates NA and PRPP. Because the addition of NA further increased NAD^+^ concentration in the Nampt-over-expressed cells, the suppressed increase in R_S_ may not be due to the severe depletion of the cellular pool of PRPP.

In addition to R_S_, our determination of R_B_ demonstrated the increased degradation of NAD^+^ in the Nampt-over-expressed cells, consistent with observations in yeast strains, where increased dosage of NPT1, the NAD^+^ salvage pathway gene, does not change cellular NAD^+^ levels [24]. Since we observed that the increase in R_B_ directly depends on that in R_S_ (Fig 6B), it is most likely that upon Nampt induction, the suppressed increase in R_S_ and the balanced increase in R_B_ contribute to the limited increase in NAD^+^ concentration in the cells (1.6-fold increase). The dependence of R_B_ on R_S_ may explain the phenomenon reported previously that more than 10-fold increases in Nampt expression elevate cellular NAD^+^ levels only 40–60% in mammalian cells and tissues over-expressing Nampt [11–14]. A particular molecular link must exist for the tight connection between the NAD^+^ synthesis and its breakdown. Our Western blot analysis of some of the NAD^+^-consuming enzymes in the Nampt-over-expressed cells excludes the induced expression of PARP1, PARP2, SIRT1, or CD38 in response to increased NAD^+^ synthesis. Further investigations will be necessary to identify the basis underlying the connection to understand the regulation of cellular NAD^+^ concentrations.

Knowing the absolute cellular concentrations of NAD^+^ is essential to evaluate the activity of NAD^+^-utilizing enzymes in cells based on *K*_m_ values for NAD^+^. However, the majority of studies have provided its cellular levels as a mole of NAD^+^ per million cells, or per mg protein or mg tissue, or sometimes only changes in its levels as fold-changes [11–14, 25–35]. Although markedly different NAD^+^ contents were observed in the mammalian cells under the resting conditions when expressed as a mole per million cells (Fig 4D), once cellular NAD^+^ concentration was calculated from the moles of NAD^+^ in the cells and their volumes measured, the NAD^+^ concentration was found to be maintained within a relatively narrow range, 400–700 μM, consistent with reported cellular NAD^+^ concentration [16], irrespective of cell origins (Fig 4I). Cellular NAD^+^ concentration obtained here could represent the concentrations of nuclear/cytosol fractions, because NAD^+^ may be readily exchangeable between the nucleus and cytoplasm [36] and mitochondria may constitute a small fraction of cellular contents in the cells we used [37, 38]. If this is the case, because the reported *K*_m_ values for NAD^+^ (shown in parentheses) [39] of NAD^+^-utilizing enzymes in the nucleus and cytoplasm, such as SIRT1 (~90 μM), SIRT2 (83 μM), SIRT6 (26 μM), PARP1 (50–100 μM), and PARP2 (130 μM), are much lower than cellular NAD^+^ concentration determined here under the resting conditions, these enzymes would not be further affected by any increase in cellular NAD^+^ concentration from the resting levels and not be involved in the increase in R_B_ upon Nampt induction described above. This notion is in markedly contrast with a widely accepted proposal that fluctuations in cellular NAD^+^ concentration are the principle regulator of SIRT1 activity [40]. SIRT1 activity might be regulated through the interaction with a regulatory protein [41] or post-translational modification [42], independent of changes in cellular NAD^+^ levels, although local collapse of NAD^+^ concentration even in the nucleus or cytoplasm cannot be excluded.

We provide the half-life of cellular NAD^+^, which is relatively long (4–12 h) and agrees with those (7–9 h) obtained from NAD^+^ flux measurement [16] as well as Nampt inhibition [43]. The relatively consistent half-life irrespective of cellular NAD^+^ concentration (Figs 5D and 6D) suggests that NAD^+^ is likely to be renewed at almost a constant rate in the cells, irrespective of its cellular levels. Since the synthesis of NAD^+^ is a process consuming a large amount of ATP [44], there may be a physiological meaning yet to be elucidated for such a constant renewal of cellular NAD^+^ without any changes in its concentrations.

Stable isotope-labeling experiments combined with mass spectrometry have been utilized for studying specific metabolic pathways, providing valuable information as to flows of metabolites in the pathways [45, 46]. Using this method, two recent studies have reported the rate of NAD^+^ turnover with high and non-physiological concentrations of d4-Nam (300 and 32 μM, respectively) [15, 16]. In the present study, we applied this method under the physiological conditions and provide requirements for accurate measurement of the rates of NAD^+^ synthesis and breakdown. We confirmed almost similar bioavailability of d4-Nam and d3-Nam to d0-Nam for NAD^+^ synthesis, and that of the d3-NAD^+^ to unlabeled NAD^+^ for degradation by PARPs. Because maximum rates of the synthesis and breakdown were observed at micromolar concentrations of Nam in the medium and thus physiological extracellular milieu [47], we set d4-Nam concentration in the medium to 2 μM. Under conditions of such a low Nam concentration, the recycling of Nam derived from the NAD^+^ degradation for NAD^+^ synthesis may cause to underestimate the rates R_S_ and R_B_, one of the major obstacles in determining the accurate rates with labeled Nam [22]. However, because we can discriminate the precursor d4-Nam from the breakdown products of d3-NAD^+^ and d0-NAD^+^, d3-Nam and d0-Nam, respectively, according to their mass differences, by setting the incubation time without significant amounts of these breakdown products compared with the precursor added, namely 3 h, we largely circumvented the drawback encountered with the labeled Nam. Together, we conclude not only that the rates obtained in our conditions are quite accurate but also that simultaneous measurement of cellular contents of NAD^+^ and Nam in the culture medium is necessary to evaluate the validity of the rates obtained.

In conclusion, with the stable isotope-labeling method combined with LC/MS/MS and cell volume determination, we provide the absolute rates of NAD^+^ synthesis and breakdown together with NAD^+^ concentration in various mammalian cells including primary cultured cells. Our measurements indicate that cellular NAD^+^ concentration is maintained within a narrow range irrespective of cell origins under the resting conditions and only moderately increases upon Nampt induction. Quantitative analysis of the relations among NAD^+^ concentration, R_S_, R_B_, and total cellular Nampt activity in the Nampt-over-expressed cells reveals that the only moderate increase in cellular NAD^+^ concentration likely results from suppressed increase in NAD^+^ synthesis and the balanced increase in its breakdown. Many studies suggest that cellular NAD^+^ levels fall during aging and in age-related diseases such as metabolic syndrome, neurodegeneration, and cancer, and that raising the NAD^+^ levels back to normal healthy levels promotes healthy aging and delays the age-related diseases [9, 10]. Further identification of a molecular basis of the tight link between the NAD^+^ synthesis and its breakdown may provide an important cue toward the NAD^+^-boosting strategy as well as an insight into homeostasis of cellular NAD^+^ concentration.

## Supporting information

S1 Fig Calibration curves for the labeled NAD^+^ and Nam. Calibration curves were constructed for d3-NAD^+^ (A) and d3-Nam (B), and were compared with those for d0-NAD^+^ and d0-Nam. The labeled and unlabeled compounds are indicated by *circles* and *squares*, respectively.
(PDF)

S2 Fig d4-Nam and d3-Nam exhibit similar bioavailability to unlabeled Nam. (A) HepG2N cells [17] were cultured in d0-Nam-free MEM supplemented with 10 μM d4- (*squares*) or d3-Nam (*triangles*). After the incubation for the indicated times, cellular contents of d3-NAD^+^ were quantified. For determination of NAD^+^ synthesis from d0-Nam (*circles*), cellular NAD^+^ was completely replaced with d3-NAD^+^ by two consecutive pre-incubation with 10 μM d4-Nam for 2 days before the pre-labeled cells were cultured in d4-Nam-free MEM supplemented with 10 μM d0-Nam. After the incubation with d0-Nam for the indicated times, cellular contents of d0-NAD^+^ were quantified. (B) HepG2 cells incubated with 20 μM d0- or d4-Nam for 16 h were further treated with 50 μM *N*-methyl-*N’*-nitro-*N*-nitrosoguanidine for 2 h in Nam-free MEM and the amounts of Nam in the medium were quantified. *White* and *black bars* indicate the amounts of d0- and d3-Nam, respectively. Data shown are the results of duplicate determinations. (C) HepG2N cells were incubated with 10 μM d0- or d4-Nam for 6 h. ATP contents in the cells were determined by luciferin/luciferase assay [47]. Data shown are the results of duplicate determinations.
(PDF)

S3 Fig The effect of d4-Nam concentrations on RS in non-cancerous cell lines and primary cultured cells. Non-cancerous cell lines C2C12 (A) and H9c2 cells (B), and primary cultured rat cardiomyocytes (C) were incubated with d0-Nam-free MEM supplemented with 2 or 10 μM of d4-Nam for 3 h. After the incubation, d3-NAD^+^ was quantified to determine R_S_. Data shown represent the mean ± S.D. of 3–4 separate experiments.
(PDF)

S4 Fig Induced expression of Nampt in HeLa cells. (A, B) Nampt expression was induced in HeLa cells by incubating the cells with 0, 0.1, 0.2, 0.3, or 1.0 μg/mL of Dox. Raw images used for the determination of Nampt protein expression with anti-Nampt antibodies are shown. The gels were first probed to detect Nampt protein with anti-Nampt antibodies (*top*) followed by re-probed to detect actin protein (*bottom*). Data shown are the results of six separate experiments (Exp 1–6).
(PDF)

S5 Fig Effects of the Dox treatment and the presence of the empty vector on R_S_, R_B_, and cellular NAD^+^ concentration. HeLa cells were not transfected or transfected with empty pTRE-Tight together with pTet-On-Advanced vectors, and were treated in the absence or presence of 1.0 μg/mL of Dox, as indicated. As a positive control, Nampt expression was induced in HeLa cells with Nampt and pTet-On-Advanced vectors in the presence of Dox (1.0 μg/mL). R_S_, R_B_, and cellular NAD^+^ concentration were determined in these cells. Data shown represent the mean ± S.D. of 3 separate experiments.
(PDF)

S6 Fig Effects of induction of Nampt on the expression of NAD^+^-consuming enzymes. HeLa cell lysates used in Exp1-3 in S4 Fig were subjected to Western blot analysis to determine the expression of PARP1, PARP2, SIRT1, and CD38 with anti-human PARP1 monoclonal (1:1000 dilution, Santa Cruz, sc-74469), anti-human PARP2 polyclonal (1:10000 dilution, Active Motif, 39744), anti-human SIRT1 monoclonal (1:10000, Cell Signaling, #8469), and anti-human CD38 monoclonal (1:1000 dilution, Santa Cruz, sc-374650) antibodies, respectively.
(PDF)

S1 Table Parameters for SRM analysis of NAD^+^ and Nam with API3000. DP, declustering potential; FP, focusing potential; CE, collision energy; CXP, collision cell exit potential; RT, retention time; LOQ, limit of quantification. Data indicated by *asterisks* are from Yamada K, Hara N, Shibata T, Osago H, Tsuchiya M. (2006) The simultaneous measurement of nicotinamide adenine dinucleotide and related compounds by liquid chromatography/electrospray ionization tandem mass spectrometry. Anal Biochem 352:282–285.
(PDF)

S2 Table Parameters for SRM analysis of NAD^+^ and Nam with LCMS-8030. CE, collision energy; RT, retention time.
(PDF)
