# Supplementary material for: Quantitative analysis of the effects of nicotinamide phosphoribosyltransferase induction on the rates of NAD+ synthesis and breakdown in mammalian cells using stable isotope-labeling combined with mass spectrometry
Source: PLoS One. 2019 Mar 15;14(3):e0214000. doi: 10.1371/journal.pone.0214000 (PMC6420012; doi:10.1371/journal.pone.0214000)
Supplement: S1 Fig — Calibration curves were constructed for d3-NAD+ (A) and d3-Nam (B), and were compared with those for d0-NAD+ and d0-Nam. The labeled and unlabeled compounds are indicated by circles and squares, respectively. (PDF) [file pone.0214000.s001.pdf]

S1 Fig. Calibration curves for the labeled NAD^+ and Nam.

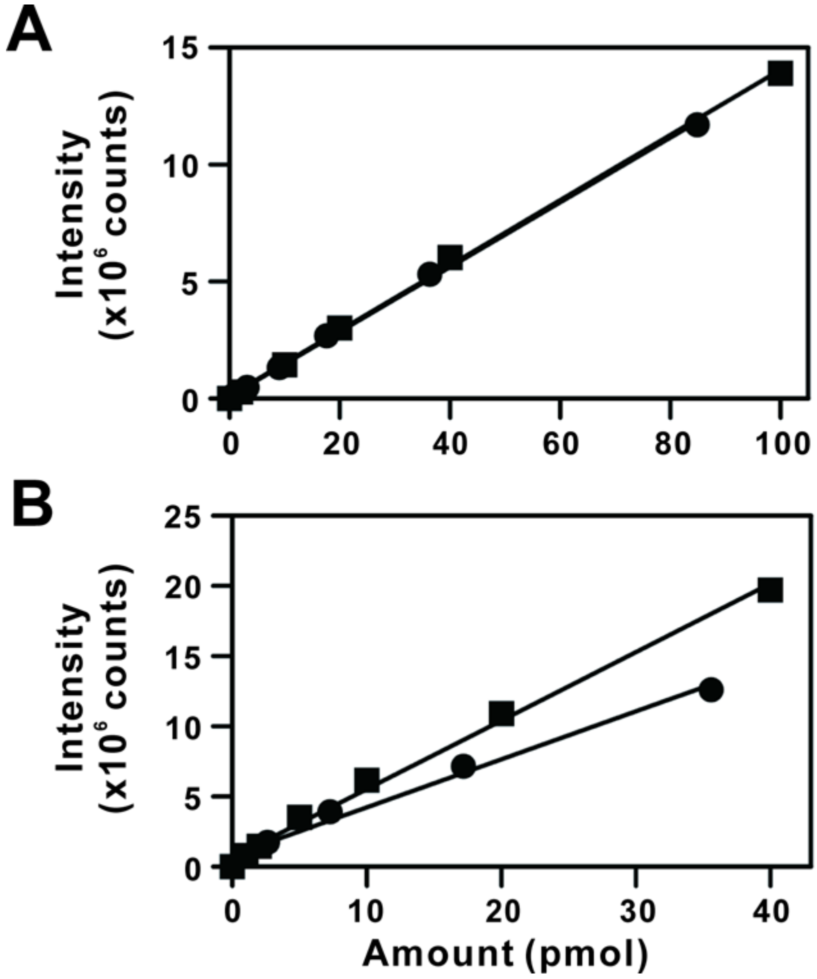

Calibration curves were constructed for d3-NAD^+ (A) and d3-Nam (B), and were compared with those for d0-NAD^+ and d0-Nam. The labeled and unlabeled compounds are indicated by *circles* and *squares*, respectively.
