# Supplementary material for: Quantitative analysis of the effects of nicotinamide phosphoribosyltransferase induction on the rates of NAD+ synthesis and breakdown in mammalian cells using stable isotope-labeling combined with mass spectrometry
Source: PLoS One. 2019 Mar 15;14(3):e0214000. doi: 10.1371/journal.pone.0214000 (PMC6420012; doi:10.1371/journal.pone.0214000)
Supplement: S2 Fig — (A) HepG2N cells [17] were cultured in d0-Nam-free MEM supplemented with 10 μM d4- (squares) or d3-Nam (triangles). After the incubation for the indicated times, cellular contents of d3-NAD+ were quantified. For determination of NAD+ synthesis from d0-Nam (circles), cellular NAD+ was completely replaced with d3-NAD+ by two consecutive pre-incubation with 10 μM d4-Nam for 2 days before the pre-labeled cells were cultured in d4-Nam-free MEM supplemented with 10 μM d0-Nam. After the incubation with d0-Nam for the indicated times, cellular contents of d0-NAD+ were quantified. (B) HepG2 cells incubated with 20 μM d0- or d4-Nam for 16 h were further treated with 50 μM N-methyl-N’-nitro-N-nitrosoguanidine for 2 h in Nam-free MEM and the amounts of Nam in the medium were quantified. White and black bars indicate the amounts of d0- and d3-Nam, respectively. Data shown are the results of duplicate determinations. (C) HepG2N cells were incubated with 10 μM d0- or d4-Nam for 6 h. ATP contents in the cells were determined by luciferin/luciferase assay [47]. Data shown are the results of duplicate determinations. (PDF) [file pone.0214000.s002.pdf]

S2 Fig. d4-Nam and d3-Nam exhibit similar bioavailability to unlabeled Nam.

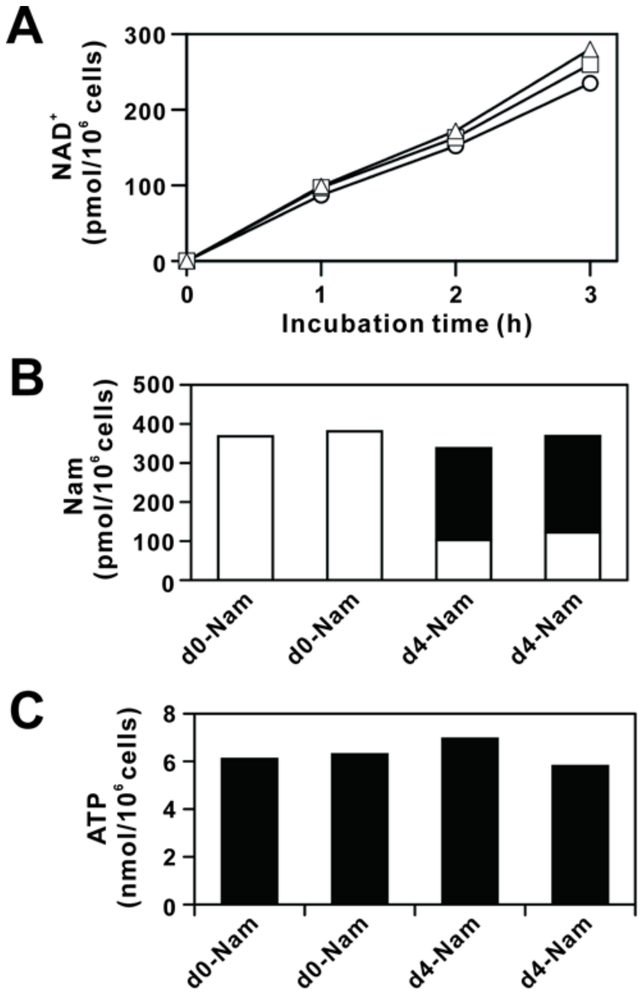

S2 Fig. d4-Nam and d3-Nam exhibit similar bioavailability to unlabeled Nam.
(A) HepG2N cells were cultured in Nam-free MEM supplemented with 10 µM d4- (*squares*), d3- (*triangles*), or d0-Nam (*circles*). After the incubation for the indicated times, cellular contents of d3-NAD$^+$ were quantified except for the incubation with d0-Nam. For determination of NAD$^+$ synthesis from d0-Nam, cellular NAD$^+$ was completely replaced with d3-NAD$^+$ by two consecutive pre-incubation with 10 µM d4-Nam for 2 days before the pre-labeled cells were cultured in Nam-free MEM supplemented with 10 µM d0-Nam. (B) HepG2 cells incubated with 20 µM d0- or d4-Nam for 16 h were further treated with 50 µM *N*-methyl-*N'*-nitro-*N*-nitrosoguanidine for 2 h in Nam-free MEM and the amounts of Nam in the medium were quantified. *White* and *black bars* indicate the amounts of d0- and d3-Nam, respectively. Data shown are the results of duplicate determinations. (C) HepG2N cells were incubated with10 µM d0- or d4-Nam for 6 h. ATP contents in the cells were determined by luciferin/luciferase assay, as described in Hara N, Yamada K, Shibata T, Osago H, Tsuchiya M.

(2011) Nicotinamide phosphoribosyltransferase/visfatin does not catalyze nicotinamide mononucleotide formation in blood plasma. PLoS One 6: e22781. Data shown are the results of duplicate determinations.
