# Supplementary material for: Quantitative analysis of the effects of nicotinamide phosphoribosyltransferase induction on the rates of NAD+ synthesis and breakdown in mammalian cells using stable isotope-labeling combined with mass spectrometry
Source: PLoS One. 2019 Mar 15;14(3):e0214000. doi: 10.1371/journal.pone.0214000 (PMC6420012; doi:10.1371/journal.pone.0214000)
Supplement: S3 Fig — Non-cancerous cell lines C2C12 (A) and H9c2 cells (B), and primary cultured rat cardiomyocytes (C) were incubated with d0-Nam-free MEM supplemented with 2 or 10 μM of d4-Nam for 3 h. After the incubation, d3-NAD+ was quantified to determine RS. Data shown represent the mean ± S.D. of 3–4 separate experiments. (PDF) [file pone.0214000.s003.pdf]

S3 Fig. The effect of d4-Nam concentrations on R_S in non-cancerous and primary cultured cells.

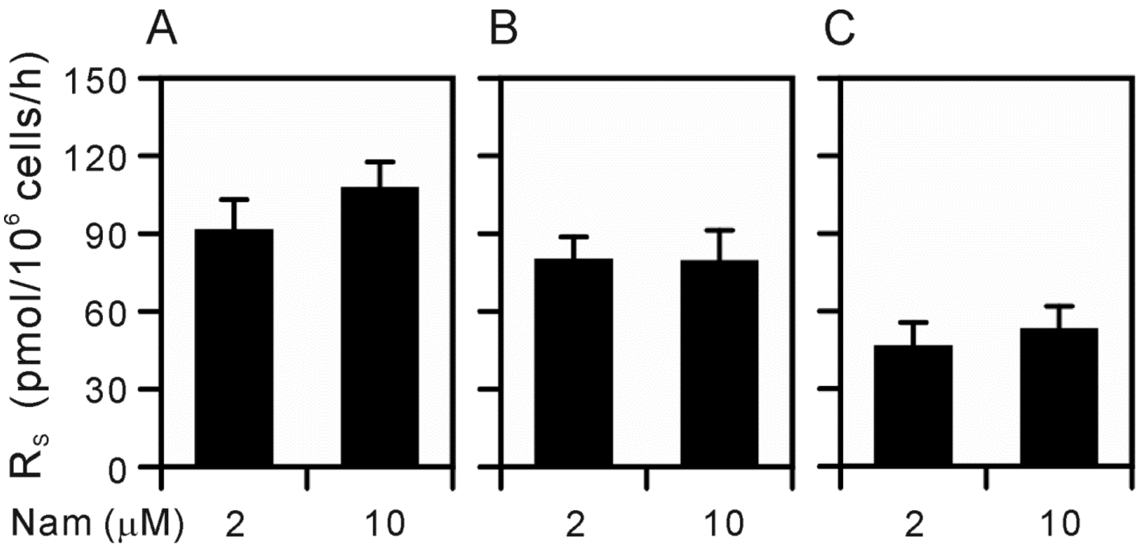

C2C12 (A), H9c2 cells (B), and rat primary cardiomyocytes (C) were incubated with d0-Nam-free MEM supplemented with 2 or 10 μM of d4-Nam for 3 h. After the incubation, d3-NAD^+ was quantified to determine R_S. Data shown represent the mean ± S.D. of 3-4 separate experiments.
