# Supplementary material for: Quantitative analysis of the effects of nicotinamide phosphoribosyltransferase induction on the rates of NAD+ synthesis and breakdown in mammalian cells using stable isotope-labeling combined with mass spectrometry
Source: PLoS One. 2019 Mar 15;14(3):e0214000. doi: 10.1371/journal.pone.0214000 (PMC6420012; doi:10.1371/journal.pone.0214000)
Supplement: S4 Fig — (A, B) Nampt expression was induced in HeLa cells by incubating the cells with 0, 0.1, 0.2, 0.3, or 1.0 μg/mL of Dox. Raw images used for the determination of Nampt protein expression with anti-Nampt antibodies are shown. The gels were first probed to detect Nampt protein with anti-Nampt antibodies (top) followed by re-probed to detect actin protein (bottom). Data shown are the results of six separate experiments (Exp 1–6). (PDF) [file pone.0214000.s004.pdf]

S4 Fig. Induced expression of Nampt in HeLa cells.

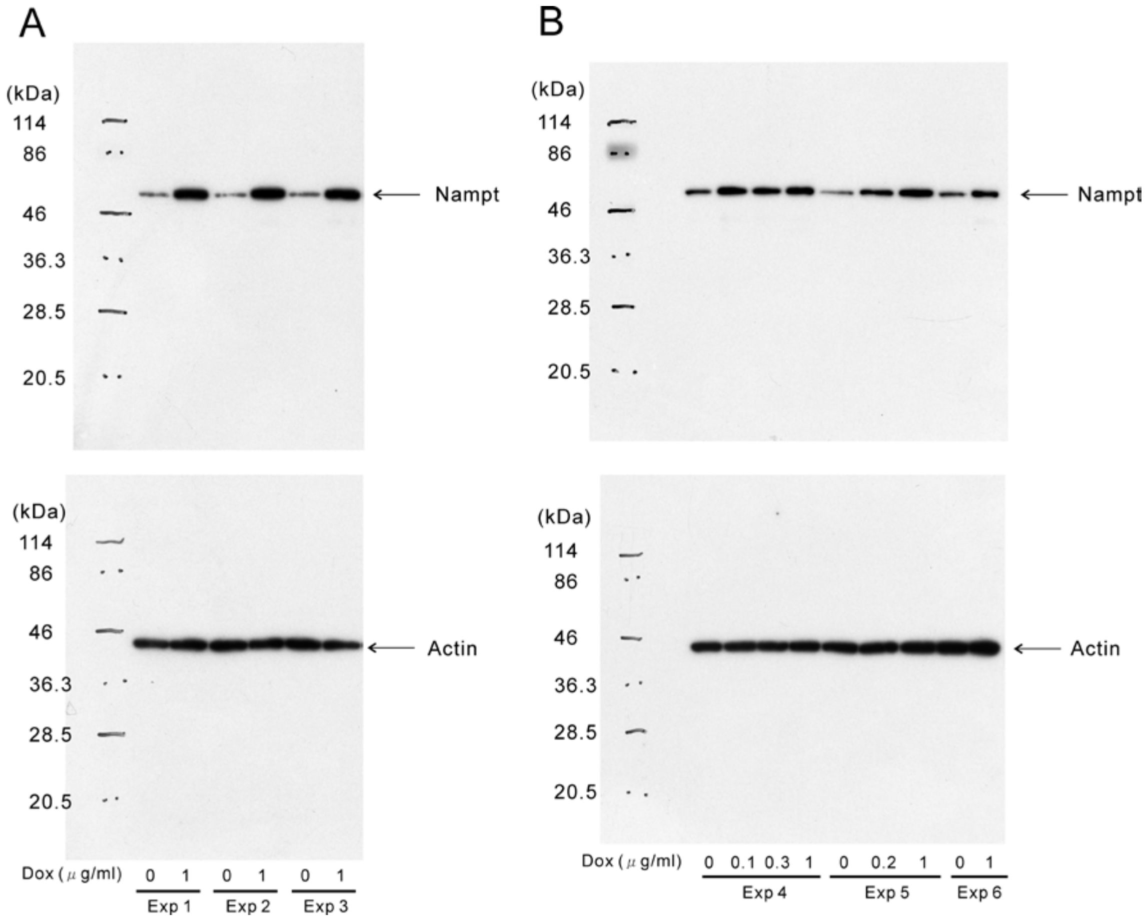

(A, B) Nampt expression was induced in HeLa cells by incubating the cells with 0.0, 0.1, 0.2, 0.3, or 1.0 µg/mL of Dox. Raw images used for the determination of Nampt protein levels with anti-Nampt antibodies. The gels were first probed to detect Nampt protein (*top*) followed by re-probed to detect actin protein (*bottom*).
