# Supplementary material for: Quantitative analysis of the effects of nicotinamide phosphoribosyltransferase induction on the rates of NAD+ synthesis and breakdown in mammalian cells using stable isotope-labeling combined with mass spectrometry
Source: PLoS One. 2019 Mar 15;14(3):e0214000. doi: 10.1371/journal.pone.0214000 (PMC6420012; doi:10.1371/journal.pone.0214000)
Supplement: S5 Fig — HeLa cells were not transfected or transfected with empty pTRE-Tight together with pTet-On-Advanced vectors, and were treated in the absence or presence of 1.0 μg/mL of Dox, as indicated. As a positive control, Nampt expression was induced in HeLa cells with Nampt and pTet-On-Advanced vectors in the presence of Dox (1.0 μg/mL). RS, RB, and cellular NAD+ concentration were determined in these cells. Data shown represent the mean ± S.D. of 3 separate experiments. (PDF) [file pone.0214000.s005.pdf]

S5 Fig. Effects of the Dox treatment and the presence of the empty vector on R_S, R_B, and cellular [NAD^+].

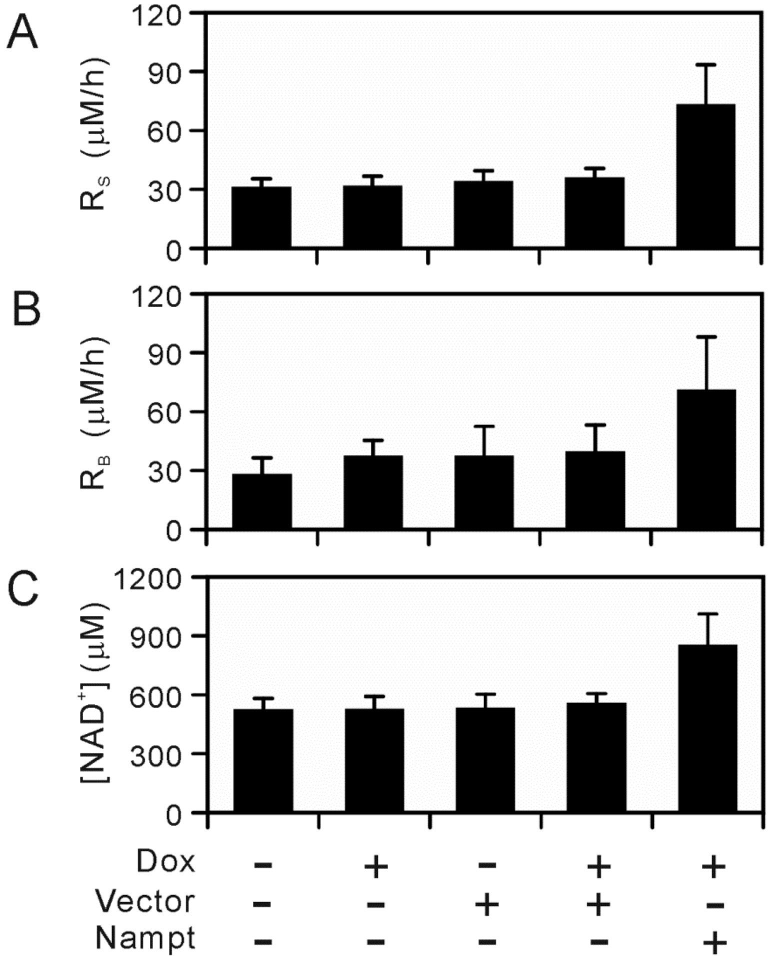

HeLa cells were not transfected or transfected with empty pTRE-Tight together with pTet-On-Advanced vectors, and were treated in the absence or presence of 1.0 µg/mL of Dox, as indicated. As a positive control, Nampt expression was induced in HeLa cells with Nampt and pTet-On-Advanced vectors in the presence of Dox (1.0 µg/mL). R_S (A), R_B (B), and cellular [NAD^+] (C) were determined in these cells. Data shown represent the mean ± S.D. of 3 separate experiments.
