# Supplementary material for: Quantitative analysis of the effects of nicotinamide phosphoribosyltransferase induction on the rates of NAD+ synthesis and breakdown in mammalian cells using stable isotope-labeling combined with mass spectrometry
Source: PLoS One. 2019 Mar 15;14(3):e0214000. doi: 10.1371/journal.pone.0214000 (PMC6420012; doi:10.1371/journal.pone.0214000)
Supplement: S6 Fig — HeLa cell lysates used in Exp1-3 in S4 Fig were subjected to Western blot analysis to determine the expression of PARP1, PARP2, SIRT1, and CD38 with anti-human PARP1 monoclonal (1:1000 dilution, Santa Cruz, sc-74469), anti-human PARP2 polyclonal (1:10000 dilution, Active Motif, 39744), anti-human SIRT1 monoclonal (1:10000, Cell Signaling, #8469), and anti-human CD38 monoclonal (1:1000 dilution, Santa Cruz, sc-374650) antibodies, respectively. (PDF) [file pone.0214000.s006.pdf]

S6 Fig. Effects of induction of Nampt on the expression of NAD^+-consuming enzymes.

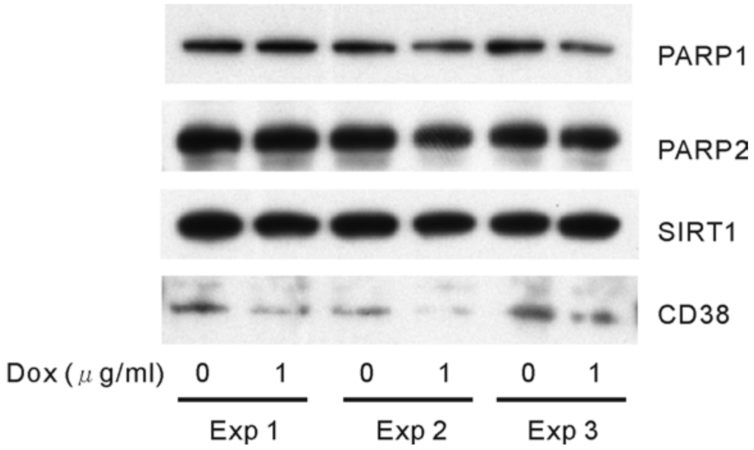

HeLa cell lysates used in Exp1-3 in S4 Fig were subjected to Western blot analysis to determine the expression of PARP1, PARP2, SIRT1, and CD38 with anti-human PARP1 monoclonal (1:1000 dilution, Santa Cruz, sc-74469), anti-human PARP2 polyclonal (1:10000 dilution, Active Motif, 39744), anti-human SIRT1 monoclonal (1:10000, Cell Signaling, #8469), and anti-human CD38 monoclonal (1:1000 dilution, Santa Cruz, sc-374650) antibodies, respectively.
