# Supplementary material for: Quantitative analysis of the effects of nicotinamide phosphoribosyltransferase induction on the rates of NAD+ synthesis and breakdown in mammalian cells using stable isotope-labeling combined with mass spectrometry
Source: PLoS One. 2019 Mar 15;14(3):e0214000. doi: 10.1371/journal.pone.0214000 (PMC6420012; doi:10.1371/journal.pone.0214000)
Supplement: S1 Table — DP, declustering potential; FP, focusing potential; CE, collision energy; CXP, collision cell exit potential; RT, retention time; LOQ, limit of quantification. Data indicated by asterisks are from Yamada K, Hara N, Shibata T, Osago H, Tsuchiya M. (2006) The simultaneous measurement of nicotinamide adenine dinucleotide and related compounds by liquid chromatography/electrospray ionization tandem mass spectrometry. Anal Biochem 352:282–285. (PDF) [file pone.0214000.s007.pdf]

S1 Table. Parameters for SRM analysis of NAD^+ and Nam with API3000

Metabolite	Transition		Parameter				RT (min)	LOQ (pmol)
	Q1	Q3	DP	FP	CE	CXP		
d0-Nam	123.0*	80.0	36	210	29	4	10.5	0.4
d3-Nam	126.0	83.1						0.2
d4-Nam	127.0	84.1						0.2
d0-NAD^+	664.2*	136.0*	42	220	67	8	8.9	0.1*
d3-NAD^+	667.2	136.0						0.1

DP, declustering potential; FP, focusing potential; CE, collision energy; CXP, collision cell exit potential; RT, retention time; LOQ, limit of quantification. Data indicated by *asterisks* are from Yamada K, Hara N, Shibata T, Osago H, Tsuchiya M. (2006) The simultaneous measurement of nicotinamide adenine dinucleotide and related compounds by liquid chromatography/electrospray ionization tandem mass spectrometry. Anal Biochem 352:282-285.
