# Supplementary material for: Quantitative analysis of the effects of nicotinamide phosphoribosyltransferase induction on the rates of NAD+ synthesis and breakdown in mammalian cells using stable isotope-labeling combined with mass spectrometry
Source: PLoS One. 2019 Mar 15;14(3):e0214000. doi: 10.1371/journal.pone.0214000 (PMC6420012; doi:10.1371/journal.pone.0214000)
Supplement: S2 Table — CE, collision energy; RT, retention time. (PDF) [file pone.0214000.s008.pdf]

S2 Table. Parameters for SRM analysis of NAD^+ and Nam with LCMS-8030

Metabolite	Transition		Parameter			RT (min)
	Q1	Q3	Q1 pre bias (V)	CE	Q3 pre bias (V)	
d0-Nam	123.1	80.1	−12	−23	−30	7.7
d3-Nam	126.2	83.1	−13	−24	−30	
d4-Nam	127.2	84.2	−13	−23	−15	
d0-NAD^+	664.1	136.1	−30	−55	−24	6.7
d3-NAD^+	667.0	136.0		−40	−22	

CE, collision energy; RT, retention time.
